# Passive intraoperative motor fMRI under general anesthesia during open-craniotomy glioma surgery: a prospective technical feasibility study

**DOI:** 10.1016/j.bas.2026.107446

**Published:** 2026-09-22

**Authors:** Jonathan Wais, Julia Shawarba, Vitalij Zeiser, Gilbert Hangel, Philipp Lazen, Georg Widhalm, Florian Ph.S. Fischmeister, Ammar Mallouhi, Gregor Kasprian, Karl Roessler

**Affiliations:** a Dept. of Neurosurgery, Medical University of Vienna, Vienna, Austria; b Dept. of Neurosurgery and Highfield MR Centre, Medical University of Vienna, Vienna, Austria; c Dept. of Neuroradiology, Medical University of Vienna, Vienna, Austria

**Keywords:** Passive functional MRI, Intraoperative MRI, General anesthesia, Sensorimotor mapping, Glioma surgery, Diffusion tensor tractography

## Abstract

**Introduction:**

Sensorimotor localization is important during peri-Rolandic glioma surgery. We assessed passive intraoperative motor functional MRI (pifMRI) under general anesthesia.

**Research question:**

Can pifMRI provide useable sensorimotor maps before and after resection for neuronavigation and comparison with cortical stimulation?

**Material and methods:**

Eight adults (24–72 years; median, 57.5) underwent pifMRI intraoperatively in a two-room 3-T intraoperative MRI setting. With cranium open, blood oxygen level-dependent signals were elicited by passive flexion-extension of digits II–V. Maps were integrated into neuronavigation and used for corticospinal tract tractography. Given the exploratory sample and absent systematic coordinate-level recording, analyses were descriptive.

**Results:**

Useable activation in the primary sensorimotor region was obtained in all patients. Stimulation at pifMRI-defined regions elicited motor responses in all patients. However, negative sites and coordinate-level distances were not recorded, precluding diagnostic-accuracy and distance analyses. Post-resection pifMRI was obtained in seven patients. Median tumor volume decreased from 17.05 to 2.78 cm^3^; median extent of resection was 84.5% (range, 44%–100%). Six patients developed new or worsened deficits; five recovered fully and one improved by 3 months. All five patients with preoperative focal motor seizures were Engel Ia at 3 months, an uncontrolled observation not attributable to pifMRI. Mean imaging time was 48 min 45 s; each pifMRI acquisition lasted about 5 min.

**Discussion and conclusions:**

This prospective series supports the feasibility of acquiring and integrating pifMRI during glioma surgery, including after resection. Correspondence with positive stimulation sites is hypothesis-generating with no quantitative validation of mapping accuracy or clinical efficacy.

## Highlights

• Passive 3-T fMRI mapped sensorimotor activation under general anesthesia.
• Activation remained detectable in all seven post-resection acquisitions.
• Positive stimulation responses occurred at sampled pifMRI-displayed sites.
• pifMRI-derived regions supported neuronavigation and CST tractography.
• Each pifMRI acquisition required about 5 min within the ioMRI workflow.

## 1 Introduction

During surgery for lesions in peri-Rolandic regions, patient-specific localization of the sensorimotor cortex may support operative planning (Roessler et al., 2005). Functional MRI (fMRI) delineates functional neuroanatomy through blood oxygen level-dependent (BOLD) signal changes associated with neural activity (Duffau, 2005; Satterthwaite and Davatzikos, 2015; Glover, 2011; Howseman and Bowtell, 1999; Sutton et al., 2009).

Although intraoperative electrocortical stimulation (ECS), with or without awake craniotomy and particularly within the motor cortex area, remains the gold standard for cortical surface mapping (Ojemann, 1993; Ghazanwy et al., 2014; Wada and Rasmussen, 2007), several studies have explored the feasibility of externally guided passive sensorimotor fMRI tasks (Gasser et al., 2005a, 2005b). Moreover, 3 T fMRIs have demonstrated comparable BOLD activation in identical anatomical regions during active and passive motor tasks in healthy awake participants (Blatow et al., 2011). Additionally, passive stimulation performed intraoperatively under general anesthesia (GA) has likewise been shown to elicit BOLD responses comparable to those obtained with awake BOLD-fMRI (Yamamoto et al., 2019).

Intraoperative structural MRI permits assessment of residual tumor and updating of neuronavigation after resection-related anatomical change (Tomschik et al., 2023). Adding passive sensorimotor fMRI (pifMRI) could provide updated functional information within the same workflow. Whether such information improves resection or clinical outcomes, however, cannot be determined without a comparator group and quantitative validation.

This prospective exploratory study investigated the technical feasibility of passive sensorimotor fMRI in a 3-T intraoperative MRI setting under general anesthesia and with an open cranium during surgery for gliomas involving or adjacent to the sensorimotor cortex. Prespecified feasibility endpoints were acquisition of anatomically plausible sensorimotor BOLD activation, integration into neuronavigation, use as a seed for diffusion tensor imaging (DTI)-based corticospinal tract (CST) tractography, and descriptive spatial correspondence with positive intraoperative cortical stimulation. The study was not designed to test clinical efficacy or diagnostic accuracy.

## 2 Methods

### 2.1 Patients

Adult patients (age: >18 years) participated in this prospective study, all of whom gave informed consent prior to inclusion. This trial was approved by the institutional ethics-committee (EK Nr: 1668/2022). The patient characteristics can be seen in Table 1. Patient-selection was based on our inclusion criteria:

a) Cerebral mass lesion within or in proximity to the sensorimotor cortex scheduled for neurosurgical resection. The outline for proximity was defined as either a mass directly located in the sensorimotor cortex or directly adjacent, judged by perifocal edema/infiltration-zone on T2/FLAIR MRI imaging.
b) Surgery was planned in the intraoperative-MRI-theater while patients having no contraindication for a magnetic field of 3T.
c) No high anesthesiologic risk factors (e.g.: significant pulmonary- and cardiovascular comorbidities) to prevent complications by prolonged operating time due to pifMRI scans under GA.

**Table 1:** **Patient-Characteristics:** Summarizes patient characteristics including gender (m/f), age (years), lesional hemisphere (left/right), tumor volume (cm^3^), extent of resection and histopathology. It also details previous neurosurgical procedures, pre- and postresection neurological symptoms, and lists the anesthesiologic agents with corresponding doses administered during pifMRI, alongside the total intraoperative MRI duration.

Patients	A	B	C	D	E	F	G	H
Gender (m/f)	f	m	f	f	f	m	f	m
Age (years)	27	65	24	69	54	61	39	72
Hemisphere (right/left)	left	right	right	right	right	left	right	right
Histology	Diffuse Astrocytoma IDH1-mut. WHO 3	Diffuse Astrocytoma IDH1-wt. WHO 4	Angiocentric Glioma WHO 1	Diffuse Astrocytoma IDH1-wt. WHO 4	Diffuse Astrocytoma IDH1-wt. WHO 4	Diffuse Astrocytoma IDH1-wt. WHO 4	Diffuse Astrocytoma IDH1-wt. WHO 4	Diffuse Astrocytoma IDH1-wt. WHO 4
Neurological Symptoms: preOP	none	CN VII: central palsy left	none	UE + central CN VII: palsy left, sensory deficit	Mild UE + LE palsy left	none	CN VII: central palsy left	CN VII: central palsy left
Neurological Symptoms: postOP	Hemiparesis: UE + LE right Transient	Hemiparesis UE + LE, central CN VII palsy left Transient	Fine motor deficit UE left Transient	Fine motor deficit UE left Transient	Mild UE + LE palsy left Transient	mild speech difficulties Transient	Unchanged	none
Seizures preOP	Yes	Yes	Yes	No	Yes	No	Yes	No
Seizures postOP	Engel Ia	Engel Ia	Engel Ia	n/a	Engel Ia	n/a	Engel Ia	n/a
Previous Surgery	none	none	Ipsilateral biopsy	none	Ipsilateral resection	none	Contralateral resection	none
Tumor Vol. (cm3) preOP	44,3	13,4	2,16	20,3	16,5	6,33	17,6	49,1
Tumor Vol. (cm3) postOP	6,82	0	0,05	10	2,7	2,79	9,93	2,76
Extent of Resection	85%	100%	98%	51%	84%	56%	44%	94%
Second-Look Surgery	Yes	No	No	No	No	No	No	No
Anesthesia – pifMRI								
Propofol	8,13 mg/kg/h	5,5 mg/kg/h	5,93 mg/kg/h	5,8 mg/kg/h	5,7 mg/kg/h	5,5 mg/kg/h	5,6 mg/kg/h	5,1 mg/kg/h
Remifentanil	0,423 μg/kg/min	0,256 μg/kg/min	0,51 μg/kg/min	0,556 μg/kg/min	0,524 μg/kg/min	0,5 μg/kg/min	0,322 μg/kg/min	0,366 μg/kg/min
Time: ioMRI (min)	48	43	42	54	59	37	40	67

Legend.

CN VII: Cranial Nerve VII.

f:Female.

IDH: Isocitrate-Dehydrogenase.

LE: Lower Extremity.

m: Male.

n/a:Not applicable.

UE:Upper Extremity.

WHO: World Health Organization.

The trial was conducted at the General Hospital of Vienna, Medical University of Vienna (Departments of Neurosurgery, Division of Neuroradiology and Musculoskeletal Radiology, Department of Biomedical Imaging and Image Guided Therapy, Medical University Vienna). All patients underwent routine supratentorial craniotomy and mass-lesion resection under GA using intraoperative 3T MRI, intraoperative electrophysiological monitoring (IOM), consisting of ECS, phase-reversal, motor evoked potentials (MEPs), somatosensory evoked potentials (SSEPs), and neuronavigation guidance (Brainlab, Munich, Germany). Each patient received a routine preoperative structural MRI (sMRI), acquired on a 3T Philips Ingenia Elition X (Philips Medical Systems, Hamburg, Germany), including T1-weighted (without and with contrast), T2-weighted, FLAIR, and DTI. Preoperative structural MRI was obtained within one week prior to scheduled surgery at the Division of Neuroradiology and Musculoskeletal Radiology, Department of Biomedical Imaging and Image Guided Therapy, Medical University Vienna. If feasible, a routine task-based motor fMRI (tb-fMRI) within the same session was obtained using a standard active motor paradigm, featuring contralateral finger-tapping with a 5 × 30 s rest block/5 × 30 s activation block design (total 5 min).

The MRI operating theater comprises a standard operating room and an adjacent 3-T MRI suite (Siemens Skyra, Siemens Healthineers, Munich, Germany), allowing direct transfer under general anesthesia. Intraoperative structural MRI was used for residual-tumor assessment and neuronavigation re-registration after anatomical changes caused by surgery. PifMRI was acquired to update the displayed location of sensorimotor activation. The pifMRI maps were available to the operating team and were compared topographically with positive intraoperative cortical stimulation findings; assessors were not blinded. Stimulation coordinates, distances from activation peaks, and a systematic set of negative stimulation sites were not prospectively stored. Consequently, diagnostic-accuracy measures could not be calculated. Extent of resection (EOR) was determined from MRI obtained within 72 h and classified as gross-total (100%), near-total (90%-99%), subtotal (50%-89%), or biopsy only ( ≤ 50%).

A standard anesthesia protocol was used, with intravenous propofol, rocuronium, and continuous remifentanil for induction and propofol-remifentanil total intravenous anesthesia for maintenance. Maintenance doses were adjusted by the neuroanesthesiology team according to clinical requirements; the study did not mandate dose changes. Propofol maintenance rates during intraoperative MRI are reported in mg/kg/h and remifentanil rates in microg/kg/min (Table 1). A radial arterial line was placed contralateral to the stimulated hand to allow passive movement. Postoperative care followed institutional protocols.

### 2.2 Statistical analysis

Because this exploratory feasibility cohort comprised eight heterogeneous patients and had no control group, only descriptive statistics were performed. Continuous variables are reported as median and range or mean and range where appropriate; categorical variables are reported as counts and percentages. No inferential testing or clinical efficacy analysis was undertaken.

### 2.3 Passive fMRI protocol

Intraoperative pifMRI under general anesthesia took place on a 3T Siemens Skyra (Siemens Healthineers, Munich, Germany). Patients were positioned supine on a Maquet Magnus table (Getinge-Maquet, Göteborg, Sweden) while the head was fixed with polyether-ether-ketone pins (Brainlab, Munich, Germany) to avoid metallic artifacts. An 8-channel NORAS head coil (LUCY; NORAS MRI products, Höchberg, Germany) was used. PifMRI was acquired, if possible, twice, before and after resection. EPI parameters were: voxel size = 3 × 3 × 3 mm^3^; TR = 2000 ms; TE = 30 ms; flip angle = 66°; matrix = 64×64; FOV = 192 mm; 54 slices; slice thickness = 3 mm. Before MR acquisition, arm positioning was checked for passive stimulation access. A passive sensorimotor paradigm consisting of passive flexion and extension of digits II–V was performed under monitor-guided control of activation and rest periods, using a block design of five 30sec rest blocks and four 30sec activation blocks plus an 11sec synchronization period between the MRI scanner and the guiding monitor (total acquisition time 4 min 41 s), with all pifMRI tasks executed by a single clinician to minimize variability (Fig. 1) (see Fig. 2).

**Fig. 1:**
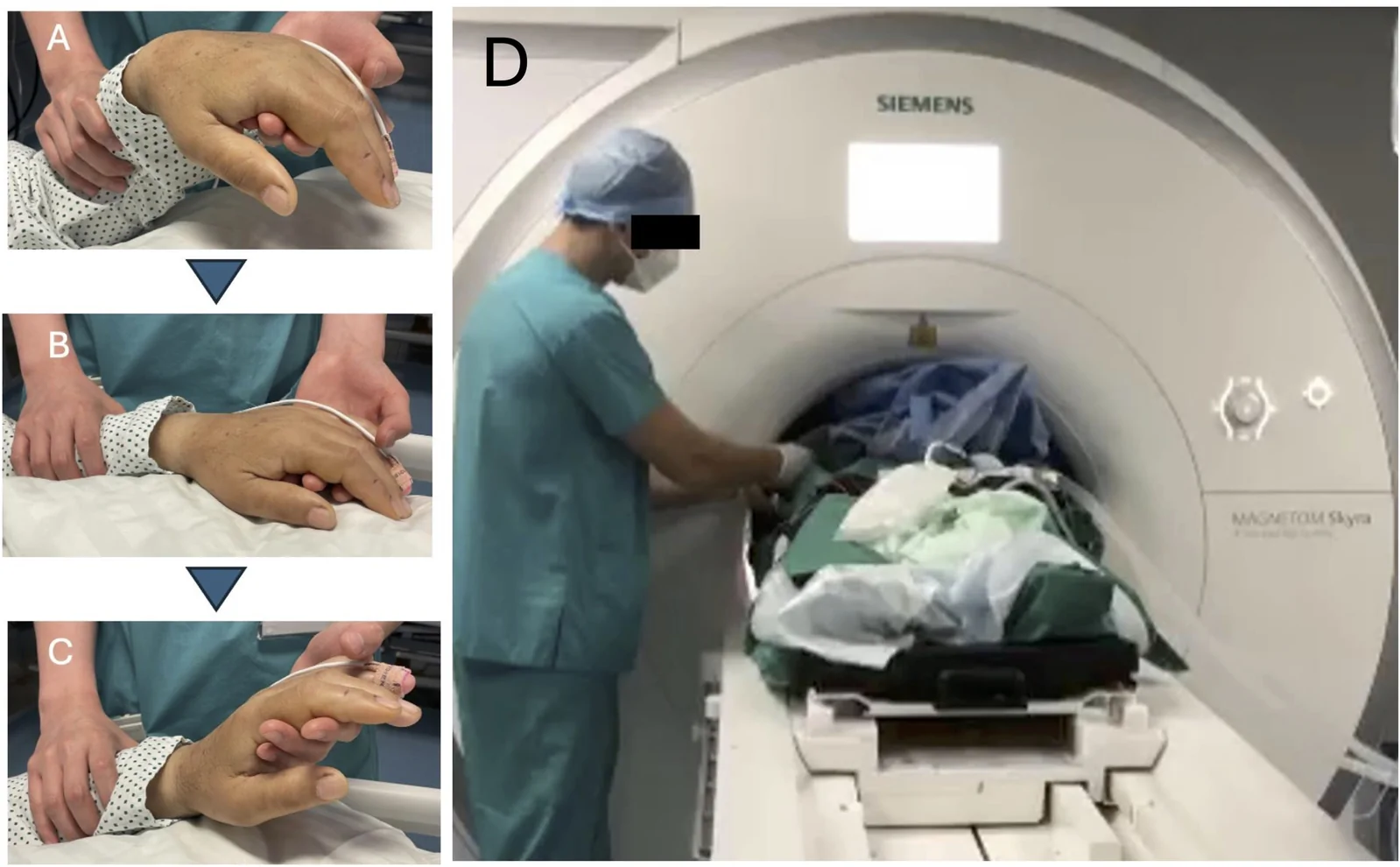
Passive stimulation by an examiner. (A) Patient’s digits II-V flexed; (B) neutral; (C) extended. During stimulation, flexion and extension alternate at 1Hz; during rest, the hand remains neutral (B). Intraoperative pifMRI acquisition with anesthetized patient with open craniotomy sterile draped. Stimulation- and resting-periods were coordinated with a monitor next to the MRI (D).

**Fig. 2:**
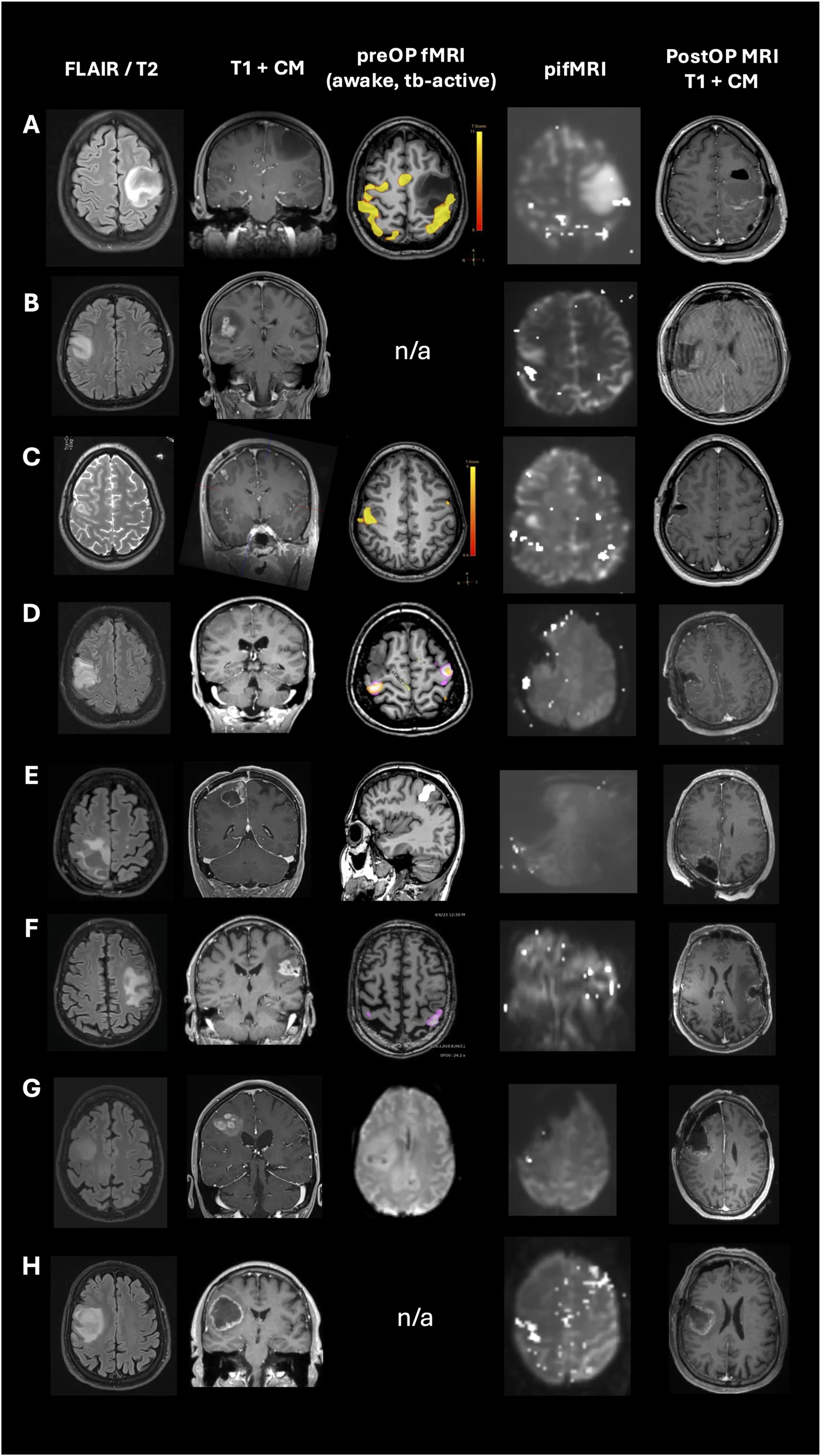
Structural MRI, awake tb-fMRI, pifMRI and post resection MRI scans for each patient (rows A–H). The first column shows axial 3T T2/FLAIR images (radiological convention) depicting the cerebral lesion. The second column displays coronal 3T T1-weighted images with gadolinium contrast-medium (CM). The third and fourth columns illustrate fMRI and pifMRI activation clusters in the sensorimotor cortex, respectively. Postoperative MRI scans can be seen in the last row (T1+CM). For Patients B and H, preoperative awake tb-fMRI was unavailable (n/a) due to organizational reasons. CM: contrast medium. n/a: Not available.

The EPI parameters were as specified above. Processing used the Brainlab BOLD MRI Mapping application (Brainlab, Munich, Germany), with fusion to high-resolution intraoperative 3-T T1-MPRAGE images. A senior neurosurgeon, aware of the operative and stimulation findings, selected an individual display threshold that isolated an anatomically plausible contralateral peri-Rolandic activation cluster relevant to the approach; the observed thresholds are reported in the Results. The commercial clinical workflow did not provide a prospectively standardized fixed statistical threshold across patients, and cluster volumes, peak t values, signal-to-noise ratios, laterality indices, and inter-rater measurements were not archived. Maps were rejected if activation was absent, predominantly extracerebral, or anatomically incompatible with the expected sensorimotor region. No such technical failure occurred in this series. Because systematic negative stimulation sites were unavailable, false-positive activation could not be formally assessed. These constraints and the operator dependence were considered part of the feasibility design and are acknowledged as limitations. Activation contours were used as cortical ROI seeds for DTI tractography, together with an ROI in the ipsilateral anterior crus cerebri. Conventional anatomical reconstructions used manual delineation of the precentral gyrus and identical tracking parameters (fractional anisotropy 0.2, fiber length ≥ 80 mm, angulation ≤ 20°). The comparison was qualitative; tract volume, overlap coefficients, Dice similarity, and tumor-to-tract distances were not prospectively recorded.

## 3 Results

Eight patients (age 24-72 years; median 57.5; five women and three men) underwent supratentorial tumor resection. Histology comprised six IDH-wildtype WHO grade 4 diffuse astrocytic tumors, one IDH-mutant WHO grade 3 diffuse astrocytoma, and one WHO grade 1 angiocentric glioma. Six lesions were right-sided, and all involved or were adjacent to the sensorimotor cortex. Five patients had preoperative neurological deficits and three had undergone prior neurosurgical procedures. Six of eight patients (75%) developed a new deficit or worsening of a pre-existing deficit immediately after surgery. By the 3-month follow-up, five of these six had recovered fully and one had improved markedly. This 75% transient neurological deterioration rate is reported descriptively and should not be interpreted as evidence of benefit. Five patients had preoperative focal motor seizures and were classified as Engel Ia at the 3-month assessment (two receiving antiseizure medication and three without); given the sample size, short follow-up, tumor heterogeneity, and absence of a comparator, no association with pifMRI was tested. Gross-total resection was achieved in one patient, near-total resection in two, subtotal resection in four, and biopsy-level resection in one. Median preoperative and postoperative tumor volumes were 17.05 cm^3^ (range, 2.16-49.1) and 2.78 cm^3^ (range, 0-10), respectively; median EOR was 84.5% (range, 44%-100%). One patient underwent additional resection after intraoperative imaging. No postoperative infection or unplanned revision surgery occurred.

All patients underwent structural MRI, and six also underwent preoperative active task-based motor fMRI. In these six cases, both active preoperative fMRI and pifMRI displayed activation centered in the expected sensorimotor region. The comparison was visual and qualitative; the active studies showed broader supplementary/premotor and contralateral co-activation. Equivalence between modalities was not assessed.

PifMRI and structural MRI under general anesthesia were performed in all eight patients. Five underwent pifMRI both before and after craniotomy/resection, two only after resection, and one only before craniotomy. In the six pre-resection acquisitions, activation was identified in the expected primary sensorimotor region at a mean operator-selected display threshold of 2.17 (range, 1.2-4.3) in the Brainlab application. Technically useable post-resection activation was present in all seven patients scanned after resection despite the altered surgical anatomy. Intraoperative neurophysiological monitoring was used in every case, and stimulation performed at the displayed pifMRI-defined cortical region elicited a motor response in all eight patients. This represents correspondence with sampled positive sites, not a measurement of sensitivity, specificity, predictive values, or localization error.

The mean intraoperative imaging time, including pre- and post-resection structural imaging for neuronavigation, surgical planning, and extent-of-resection assessment, was 48 min 45 s on average, with pifMRI accounting for 9.6% of total acquisition time in patients undergoing a single pifMRI scan (n = 3, 37.5%) and 19.2% in those undergoing two scans (n = 5, 62.5%). Mean anesthesia duration was 360 min (222–464 min), with pifMRI duration of 4 min 41s at 1.3% of total mean anesthesia time. No anesthesiologic complications occurred.

PifMRI-seeded CST reconstruction using an ipsilateral crus cerebri ROI was technically possible in all patients and produced anatomically plausible descending tracts. Post-resection datasets remained useable after the vendor software distortion-correction workflow. These observations were assessed qualitatively. No quantitative displacement, pre/post localization error, tract volume, overlap, Dice coefficient, or minimum tumor-to-tract distance was prospectively recorded; therefore, the study does not establish correction of brain shift or equivalence to anatomically seeded tractography.

## 4 Discussion

The principal finding is technical feasibility: pifMRI could be acquired under general anesthesia during open-craniotomy glioma surgery, including after resection, and could be integrated into neuronavigation and pifMRI-seeded CST tractography. At cortical regions selected from the pifMRI display, stimulation elicited motor responses in all eight patients. Because mapping was not blinded, negative stimulation sites and coordinates were not systematically stored, and imaging-quality metrics were unavailable, this observation should be regarded as descriptive spatial correspondence rather than validation of diagnostic accuracy. The cohort size, heterogeneity, and absence of a control group preclude conclusions about EOR, neurological recovery, seizure outcome, or clinical superiority (see Fig. 3, Fig. 4).

**Fig. 3:**
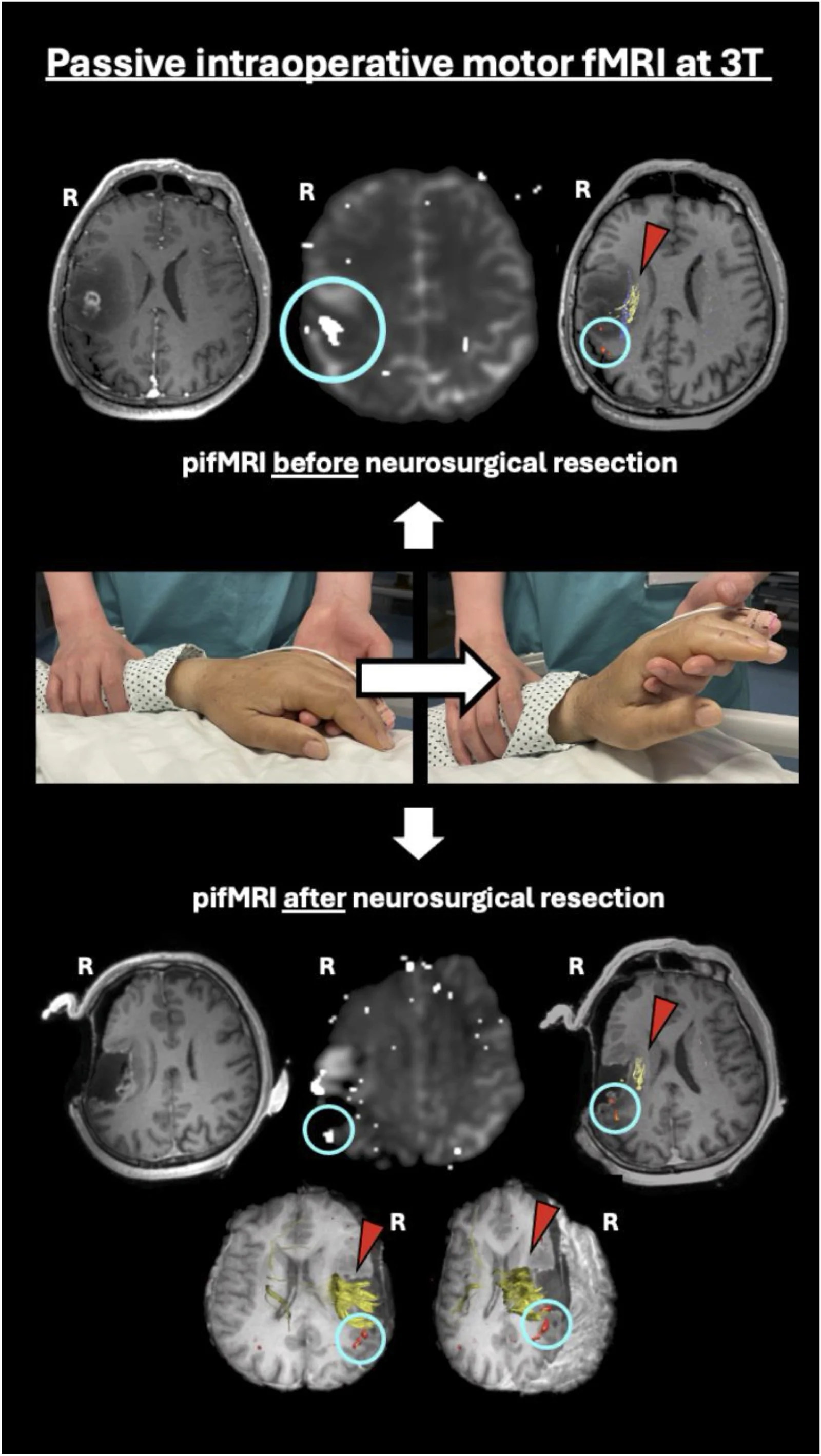
Example of pifMRI acquisition before and following craniotomy and resection (Patient B). Blue Circle: BOLD signal. Red arrow: Reconstructed fiber tract of the CST using pifMRI generated BOLD signals as ROI-seeds.

**Fig. 4:**
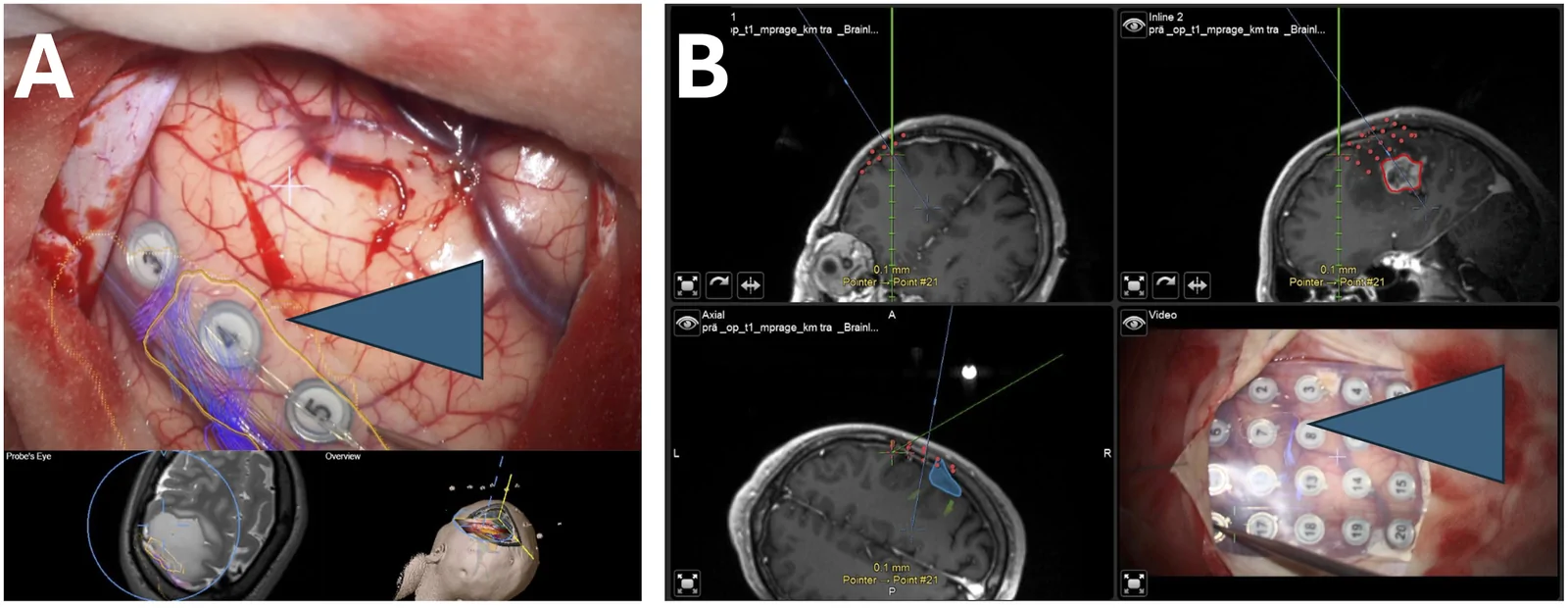
Intraoperative microscopic view with neuronavigation of Patient A (A) and Patient B (B). Blue arrowheads indicate locations at which stimulation corresponding to the displayed pifMRI BOLD activation (orange contour) elicited a motor response; associated DTI-based fiber tracts are also shown. This figure illustrates spatial correspondence at sampled positive sites and not quantitative validation.

### 4.1 PifMRI technique

Previously, it was shown that passive motor tasks on 3T MRI could yield BOLD activations comparable to active tasks (Blatow et al., 2011). This supports their use when active movement is infeasible due to deficits or poor patient compliance. Additionally, we adapted this concept to perform pifMRIs with open craniotomies during surgery in our intraoperative MRI Suite. Consequently, our 3T MRI findings even with an open craniotomy align with previous observations (Gasser et al., 2005a, 2005b; Blatow et al., 2011; Yamamoto et al., 2019)–(Gasser et al., 2005a, 2005b; Blatow et al., 2011; Yamamoto et al., 2019). Compared to awake fMRI, pifMRI yielded comparable activations in the primary sensorimotor cortex but showed differences in supplementary/premotor cortices and contralateral co-activation (Fig. 5). This indicates pifMRI primarily reflects proprioceptive signals in the central region, consistent with previous studies highlighting the limited activation of higher-order motor areas during passive tasks (Mima et al., 1999; Choudhri et al., 2015; Lee et al., 1998).

**Fig. 5:**
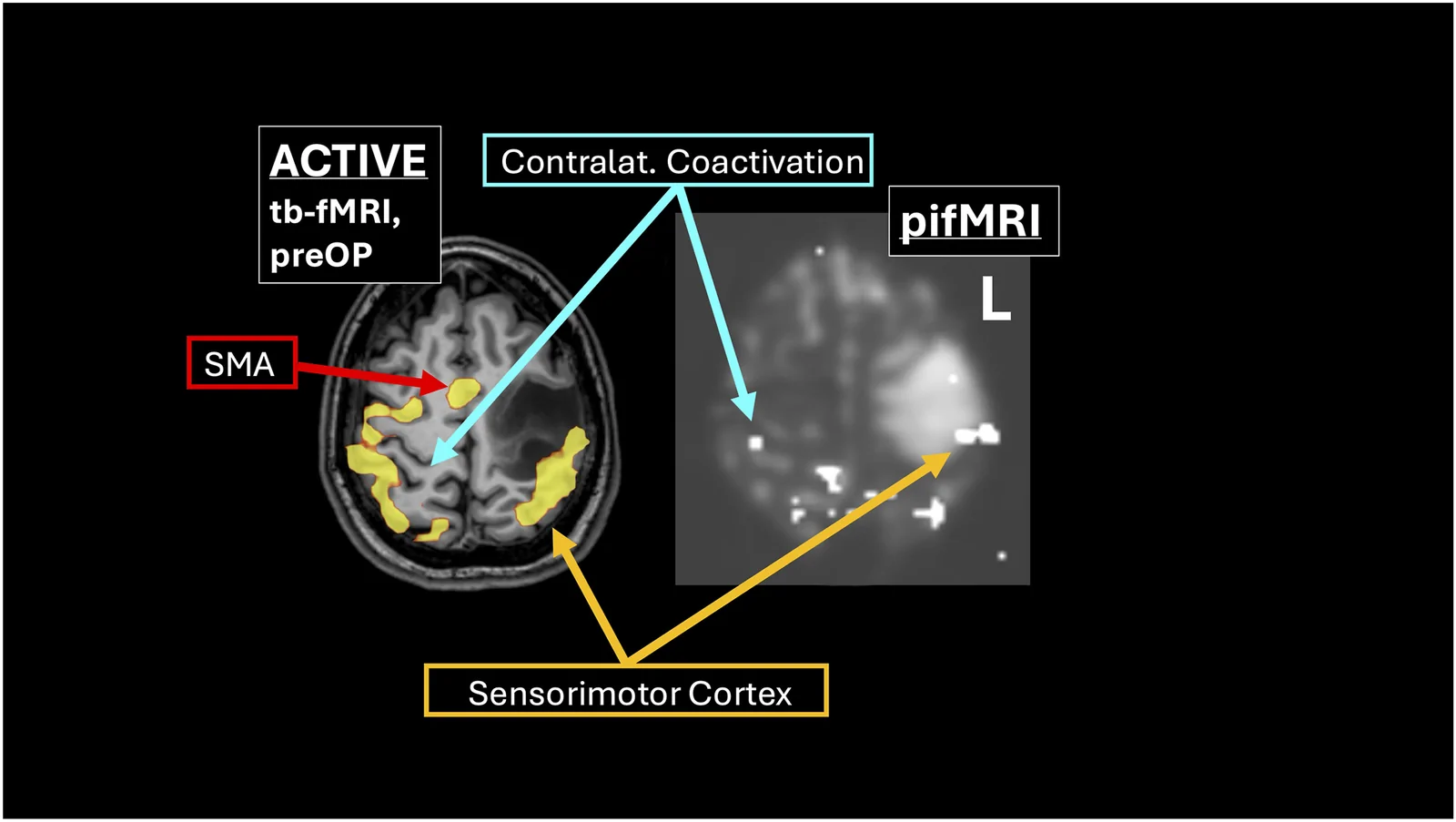
Qualitative comparison of routine preoperative active task-based fMRI and intraoperative pifMRI under general anesthesia (Patient A). Both displayed activation in the expected primary sensorimotor region; the active acquisition showed broader supplementary motor and contralateral co-activation. No quantitative equivalence analysis was performed. SMA: supplementary motor area.

Passive sensorimotor fMRI at 1.5 T under general anesthesia has previously produced clinically useful activation maps for neuronavigation (Yamamoto et al., 2019). Our 3-T observations extend this technical concept to an open-craniotomy intraoperative workflow. A uniform block design and a single examiner were used to reduce task variability. Nevertheless, anesthesia can alter the BOLD response, and the operator-selected display threshold was not standardized across patients. Quantitative BOLD-quality measures and inter-rater reliability were not available and should be included in subsequent validation studies.

Several studies have described fMRI-based ROI seeding for DTI tractography in neurosurgical planning (Kokkinos et al., 2023; Holodny et al., 2001; Sanvito et al., 2020; Smits et al., 2007). In our series, pifMRI activation could be used as an ROI seed and generated anatomically plausible CST reconstructions. This was a technical, qualitative comparison only. DTI tractography remains sensitive to acquisition and reconstruction choices, edema, crossing fibers, susceptibility effects, and postoperative distortion (Pujol et al., 2015; Azad and Duffau, 2020; Henderson et al., 2021; Roder et al., 2021). Quantitative tract overlaps and tumor-to-tract distance analyses are therefore required before equivalence to anatomical seeding or compensation for brain shift can be claimed.

PifMRI, resting-state fMRI (rs-fMRI), navigated transcranial magnetic stimulation (nTMS), and direct electrical stimulation address different parts of the mapping pathway. Rs-fMRI requires no task and may map several networks, but its analysis is less direct and generally preoperative (Manan et al., 2021; Hou et al., 2016). nTMS provides a non-invasive preoperative motor map and can support tractography, but it does not update localization after intraoperative anatomical change (Umana et al., 2021). Direct cortical and subcortical stimulation remains the operative reference standard because it tests function in real time. PifMRI is therefore proposed only as an adjunct: it provides task-linked, whole-volume imaging under general anesthesia within an existing intraoperative MRI workflow, while lacking the causal specificity and temporal resolution of stimulation.

A pifMRI acquisition lasted 4 min 41 s and could be incorporated into the intraoperative MRI session. This represented 9.6% of mean imaging time for a single acquisition. The present study did not formally evaluate workflow efficiency, cost, or whether the additional information changed decisions beyond structural MRI and electrophysiological mapping.

### 4.2 Clinical benefits of pifMRI

Extent of glioma resection is associated with survival, but surgery near the sensorimotor cortex requires an onco-functional balance (Sanai and Berger, 2008; D’Amico et al., 2017; De et al., 2021; Duffau and Mandonnet, 2013). Intraoperative imaging and electrophysiological monitoring support maximal safe resection (Magill et al., 2017; Fatemi et al., 2018). PifMRI may provide complementary patient-specific functional information, particularly when active task performance is impossible, but the contribution of pifMRI cannot be separated from these established techniques in this uncontrolled series.

Brain tumors and neurovascular uncoupling can reduce or displace BOLD activation (Holodny et al., 2000), and neurological deficits may limit conventional active tasks (Morrison et al., 2016). Despite preoperative deficits in five patients, an anatomically plausible pifMRI cluster was identified in all eight. The terms “robust” and “reproducible” are avoided because cluster volume, peak statistics, signal-to-noise ratio, laterality, and repeatability were not quantified.

Post-resection pifMRI may be the most distinctive technical observation in this series. Activation remained detectable in all seven post-resection acquisitions despite an open cranium and altered anatomy. Thresholds were adjusted individually as described in the Methods, and the standard vendor distortion-correction workflow was applied. Motion was limited by rigid head fixation and general anesthesia. Susceptibility and cavity-related artifacts were assessed visually within the clinical software; no systematic artifact score or quantitative false-positive analysis was available. In one patient, the combined intraoperative assessment prompted additional resection, but the independent contribution of pifMRI to that decision cannot be isolated retrospectively.

Stimulation at pifMRI-displayed cortical regions produced motor responses in all patients, supporting spatial correspondence at the sampled positive sites. However, the operating team was not blinded to pifMRI, the stimulation sampling strategy was clinically driven, and negative sites and three-dimensional coordinates were not systematically archived. Euclidean distances, sensitivity, specificity, and predictive values therefore cannot be reconstructed reliably. These measurements require a prospective validation protocol with blinded assessment and standardized positive and negative stimulation sampling.

The median EOR was 84.5%, and all five patients with preoperative focal motor seizures were Engel Ia at the 3-month assessment (Xu et al., 2017). These outcomes are reported to describe the cohort only. The absence of a comparator, the small and histologically heterogeneous sample, concurrent use of intraoperative structural MRI and multimodal electrophysiological monitoring, and limited seizure follow-up prevent attribution of either EOR or seizure outcome to pifMRI.

The immediate postoperative neurological deterioration rate was 75%, although five of six affected patients recovered fully and one improved markedly by 3 months. This rate should be interpreted cautiously: direct comparison with institutional benchmarks, awake mapping, or asleep motor mapping is not valid without matched case complexity, tumor topography, baseline function, and standardized outcome assessment. Recent meta-analytic evidence suggests broadly comparable neurological outcomes for awake and asleep motor mapping in selected glioma populations (Abo-elnour et al., 2024), but the present series was not designed for such a comparison. Additional limitations include selection of patients suitable for prolonged anesthesia and 3-T MRI, pathology heterogeneity, unblinded operator-dependent processing, absence of quantitative BOLD and brain-shift metrics, absence of quantitative CST comparison, and lack of an independent clinical-effect analysis.

## 5 Conclusions

In this prospective eight-patient technical feasibility study, 3-T pifMRI generated anatomically plausible sensorimotor activation during open-craniotomy glioma surgery and could be integrated into neuronavigation and CST tractography, including after resection. Positive cortical stimulation responses were observed at the pifMRI-displayed regions, but the study did not provide quantitative diagnostic validation. These findings support further prospective evaluation with standardized thresholds, quantitative BOLD and spatial metrics, blinded comparison with positive and negative stimulation sites, and a comparator group. No conclusion regarding improved EOR, neurological outcome, seizure control, brain-shift correction, or superiority to established mapping methods can be drawn from this series.

## Ethics declaration

Written informed consent to take part in the study and to publish the article has been obtained from all participants or their legal representatives. The privacy rights of participants have been observed.

This study was performed in compliance with relevant laws, regulatory frameworks and guidelines where the research took place. This study was approved by the Ethikkommission Medizinische Universität Wien. (Approval No. EK Nr: 1668/2022)

## Declaration of generative AI and AI-assisted technologies in the manuscript preparation process

During the preparation of this work the authors used Microsoft Copilot within Microsoft Word in order to help with translation from German into English if necessary. After using this tool, the authors reviewed and edited the content as needed and take full responsibility for the content of the published article.

## Declaration of competing interest

The authors declare that they have no known competing financial interests or personal relationships that could have appeared to influence the work reported in this paper.
